# Promiscuous Roles of Autophagy and Proteasome in Neurodegenerative Proteinopathies

**DOI:** 10.3390/ijms21083028

**Published:** 2020-04-24

**Authors:** Fiona Limanaqi, Francesca Biagioni, Stefano Gambardella, Pietro Familiari, Alessandro Frati, Francesco Fornai

**Affiliations:** 1 Department of Translational Research and New Technologies in Medicine and Surgery, University of Pisa, Via Roma 55, 56126 Pisa, Italy; f.limanaqi@studenti.unipi.it; 2 I.R.C.C.S. Neuromed, Via Atinense 18, 86077 Pozzilli, Italy; francesca.biagioni@neuromed.it (F.B.); stefano.gambardella@neuromed.it (S.G.); alessandro.frati@uniroma1.it (A.F.); 3 Department of Human Neurosciences, Division of Neurosurgery, Sapienza University of Rome, 00185 Roma, Italy; pietro.familiari@uniroma1.it

**Keywords:** alpha-synuclein, amyloid-beta, tau, TDP-43, SOD-1, FUS, huntingtin, prion-like, cell-to-cell propagation, neuro-inflammation

## Abstract

Alterations in autophagy and the ubiquitin proteasome system (UPS) are commonly implicated in protein aggregation and toxicity which manifest in a number of neurological disorders. In fact, both UPS and autophagy alterations are bound to the aggregation, spreading and toxicity of the so-called prionoid proteins, including alpha synuclein (α-syn), amyloid-beta (Aβ), tau, huntingtin, superoxide dismutase-1 (SOD-1), TAR-DNA-binding protein of 43 kDa (TDP-43) and fused in sarcoma (FUS). Recent biochemical and morphological studies add to this scenario, focusing on the coordinated, either synergistic or compensatory, interplay that occurs between autophagy and the UPS. In fact, a number of biochemical pathways such as mammalian target of rapamycin (mTOR), transcription factor EB (TFEB), Bcl2-associated athanogene 1/3 (BAG3/1) and glycogen synthase kinase beta (GSk3β), which are widely explored as potential targets in neurodegenerative proteinopathies, operate at the crossroad between autophagy and UPS. These biochemical steps are key in orchestrating the specificity and magnitude of the two degradation systems for effective protein homeostasis, while intermingling with intracellular secretory/trafficking and inflammatory pathways. The findings discussed in the present manuscript are supposed to add novel viewpoints which may further enrich our insight on the complex interactions occurring between cell-clearing systems, protein misfolding and propagation. Discovering novel mechanisms enabling a cross-talk between the UPS and autophagy is expected to provide novel potential molecular targets in proteinopathies.

## 1. Introduction

Alterations in the two major eukaryotic cell-clearing systems, autophagy and the ubiquitin proteasome system (UPS), are widely implicated in protein aggregation and toxicity occurring in neurodegenerative diseases. These include a progressively increasing number of neurological disorders such as Parkinson’s disease (PD), Alzheimer’s disease (AD), Huntington’s disease (HD), brain ischemia/hypoperfusion, amyotrophic lateral sclerosis (ALS) and frontotemporal dementia (FTD), among many others [1,2,3,4,5,6,7,8,9,10,11,12,13,14,15,16]. Despite etiological differences and neuroanatomical site-specificity, a failure in cell-clearing systems associated with aggregation and deposition of either misfolded or abnormally expressed proteins is emerging as a unifying feature in these disorders [7,11,14]. Examples of specific disease-associated proteins include alpha-synuclein (α-syn) in synucleinopathies such as PD and multiple system atrophy (MSA), amyloid-beta (Aβ) and tau in AD, polyglutamine-expanded huntingtin in HD, TAR-DNA-binding protein of 43 kDa (TDP-43) in ALS/FTD, as well as TDP-43, Cu/Zn superoxide dismutase (SOD), or fused in sarcoma (FUS) in ALS [12,17,18,19]. Such a concept led at first to the assumption that each disease was bound to a specific, typically misfolded protein. This was not fully confirmed since, although a certain degree of precision medicine can be drawn from the knowledge of proteinopathies, a certain amount of overlapping and non-specificity exists between aggregated proteins and various syndromes [20,21]. In fact, the concept of protein-specificity in neurodegeneration has been progressively challenged, showing that the co-existence of different proteins within aggregates may occur in either single neurological disorders or mixed neurological syndromes [14,21,22]. For instance, beyond classic synucleinopathies, α-syn aggregation occurs in ALS, AD, HD and brain ischemia, and α-syn aggregates also co-localize with TDP-43, Aβ and tau, overcoming the clear-cut distinction between degenerative and vascular dementia [23,24,25,26]. Again, FTD patients show protein inclusions which stain for either TDP-43, tau or FUS [27], while SOD1 positive inclusions have been reported in some PD cases [28]. These findings strengthen the hypothesis of a leading role of an altered proteostasis occurring independently of nosography and symptomatology across different CNS disorders.

As a further common mechanism associated with altered protein degradation in these diseases, the hypothesis of a prion-like protein propagation has been proposed [14,22,29,30,31,32,33,34,35,36]. This is supported by evidence that, similar to the prion protein and its misfolded scrapie isoform, undigested, misfold-prone proteins such as α-syn, tau, Aβ, huntingtin, SOD-1, TDP-43 and FUS can form intracellular aggregates in a self-templating manner [37,38,39,40,41,42,43,44]. In fact, these proteins possess prion-like properties, which allow spontaneous conversion of the normal isoform into abnormal ones spreading via cell-to-cell transmission [36,37,38,39,40,41,42,43,44,45,46].

This concept implies a trans-synaptic spreading based on the presence of interconnected circuitries, where misfolded proteins, upon exposure to plasma membrane, may pass from one cell to another. Recently, a deeper insight into the cell-to-cell mechanisms of communication allowed demonstration of plenty of mechanisms for disease spreading following protein misfolding. In fact, concepts which invade neuroscience coming from molecular immunohematology and oncology clearly evidence the chance that undigested cargoes of misfolded proteins can be released by the primary cell and diffuse in the extracellular space, including an entry into the bloodstream to reach distant areas [36,37,38,39,40,41,42,43,44,45,46]. In this way, a variety of structures such as exosomes can spread the disease process. In fact, misfolded proteins can spread either as free or exosome-engulfed undigested cargoes, seeding for further aggregation in recipient cells [47,48,49,50,51].

To date, plenty of evidence exists linking alterations of either autophagy or UPS with the aggregation, spreading and toxicity of so called prionoids, including α-syn, Aβ, tau, huntingtin, SOD1, TDP-43 and FUS [19,29,52,53,54,55,56,57,58,59,60]. Albeit being both autophagy and UPS implicated in the clearance of the above-mentioned proteins, recent biochemical and morphological studies have added to this scenario, focusing on the cross-talk between these two systems [61,62,63,64,65,66,67,68,69,70,71,72,73]. In fact, a coordinated, either synergistic or compensatory interplay occurs between autophagy and the UPS, which has attracted the attention of many investigators while challenging the traditional view of two segregated and independent degradation systems.

Besides sharing prion-like protein substrates, autophagy and UPS converge at both biochemical and morphological levels [61,62,63,64,65,66], though the functional significance of such an interplay remains to be fully elucidated. A growing number of biochemical pathways are being shown to operate at the crossroad between autophagy and UPS, orchestrating the specificity and magnitude of the two degradation systems for effective protein homeostasis [61,62,63,64,65,66,67,68,69,70,71,72,73,74].

In the present review, after providing an overview on such a coordinated activity between autophagy and UPS, we move to discuss evidence centered on both overlapping and divergent aspects of autophagy and UPS-dependent degradation, with a focus on prion-like proteins. This is supposed to add novel viewpoints which may further enrich our insight on the complex interactions occurring between the cell-clearing systems in protein misfolding. Since the UPS and autophagy are clearly linked to each other, understanding how alterations in one system may affect the output of another may be the key to uncovering molecular targets aimed at altering protein aggregation and propagation.

## 2. Emerging Mechanisms Underlying Autophagy and Proteasome Cross-Talk in Health and Disease

Autophagy- and UPS-dependent degradation of unfolded and misfolded proteins is important to prevent their accumulation, aggregation, and spreading [11,49,53,54,55,56,57,58,74]. Upon ubiquitination, most misfolded proteins are degraded by the proteasome 26S (P26S), which is formed by a catalytic core (P20S) and two regulatory subunits (P19S, also known as PA700) capping the ends of P20S [11,75]. P19S binds the poly-ubiquitin chain and cleaves it from the substrate, which is then unfolded and cleaved into small peptides passing through the narrow chamber of the proteasome. Here, protein degradation is eventually carried out by the β1, β2, and β5 catalytic subunits of the P20S, owning chymotrypsin-, trypsin-, and caspase-like activity, respectively [75]. UPS-dependent degradation may be restricted to soluble misfolded proteins or small oligomers that, following unfolding, are allowed to enter the P20S catalytic chamber. The latter may remain inaccessible to larger, insoluble aggregates, preventing their effective degradation [76,77]. In fact, many oligomers and aggregated proteins have been shown to inhibit UPS activity [7,55,78,79]. When the UPS is impaired, an upregulation of autophagy may occur to enable the clearance of larger aggregates [67,80]. Thus, a variety of cellular substrates, including entire organelles, as well as large aggregation-prone and/or insoluble proteins being resistant to the UPS are degraded by the autophagy machinery [81]. This occurs following substrates sequestration or shuttling into nascent, double-layered membrane vacuoles named phagophores, which then mature and seal to form the autophagosome staining for autophagy markers such as beclin-1 (the orthologue of yeast Atg6) and LC3-II (Atg8) [82]. Autophagosome cargoes are then delivered to the lysosomal compartment, which is gifted with a rich enzymatic activity. The merging of the autophagosome with endosomes (including multivesicular bodies) and lysosomes generates the catalytic organelle autophagolysosome, where cargo degradation and recycling occurs [83].

Several proteins operate at the crossroad between UPS and autophagy to regulate the sorting and shuttling of ubiquitinated substrates towards either system. For instance, ubiquitin tagging is key in sorting protein substrates for either UPS- or autophagy-dependent degradation [84]. In this context, ubiquilin proteins (UBQLNs) bind the ubiquitin chains which are attached to a variety of aggregation-prone proteins, fostering their delivery and degradation by either UPS or autophagy [85,86,87]. Dysregulated UBQLNs are associated with an impaired protein degradation by both UPS and autophagy in models of neurodegeneration [88]. A similar role is played by Parkin, an E3 ubiquitin-ligase, which produces protein ubiquitination and serves as a signal for targeting misfolded proteins to the aggresome, where autophagy is recruited [89,90]. Parkin-dependent ubiquitination triggers the removal of the pro-apoptotic proteins BAX and BCL-2 by either UPS or autophagy, and it is seminal to induce mitophagy, that is, mitochondria-specific autophagy [89,90]. After ubiquitin linkage, Parkin induces the coupling of target proteins with dynein motor complexes via the adaptor protein histone deacetylase 6 (HDAC6) in order to facilitate their transport to the aggresome, where autophagy occurs [91]. HDAC6 is a microtubule-associated histone deacetylase, which upon binding the polyubiquitin chains or even C-terminal regions of free ubiquitin, shuttles polyubiquitinated substrates along the microtubules for autophagosome engulfment, while fostering lysosome transport to the site of autophagy occurrence [92,93,94]. HDAC6 activity is essential for autophagy to compensate for protein degradation and rescue cell survival when UPS is impaired [95], providing a functional link between autophagy and UPS.

Finally, sequestosome 1 (SQSTM1)/p62 is an ubiquitin-binding scaffold protein that links ubiquitinated proteins to either UPS or autophagy [66,96,97]. On the one hand, the shuttling of ubiquitinated substrates to the autophagy machinery is due to a direct interaction between SQSTM1/p62 and ubiquitinated proteins via a C-terminal UBA domain. This in turn mediates their binding to autophagy proteins such as LC3 and GABARAP family proteins [66]. As p62 is itself degraded by autophagy, it is widely used as a marker of autophagy flux [98]. On the other hand, Phox and Bem1p (PB1) domain facilitates p62 ability to bind the UPS, thereby shuttling soluble ubiquitinated substrates for UPS degradation [96].

Besides operating mainly in the cytosol, the UPS also associates with vesicular organelles, including Golgi-derived vesicles, mitochondria, precursor synaptic vesicles, lysosomes and also autophagy-like vacuoles [63,64,99]. Such a morphological co-localization of UPS with membrane-limited intracellular compartments underlies a functional cooperation aimed at ensuring the effectiveness of endocytic, secretory, trafficking and degradation pathways. In fact, while vacuolar organelles may serve as a ferryboat to shuttle UPS back and forth among synapses, axons and cell bodies, the UPS handles the turnover of vesicle-associated proteins. This is also key for a variety of synaptic, aggregation-prone proteins which are suggested to spread cell alterations from synapses to cell bodies [100,101]. In this scenario, it is conceivable that the convergence of UPS and autophagy may empower protein homeostasis.

In keeping with this, recent studies characterized a novel organelle where autophagy and UPS markers co-localize, which is known as “autophagoproteasome” [63,64]. The merging of autophagy and UPS within such a specific autophagy-like compartment is hindered by the administration of the neurotoxic, abused drug methamphetamine (METH), which produces dopamine-related behavioral and structural alterations, including intracellular inclusions staining for ubiquitin, α-syn and also prion protein [64,102,103]. Remarkably, inhibition of the mammalian target of rapamycin (mTOR) reverses METH-induced alterations, and in particular, it rescues the merging of autophagy and UPS while enhancing cell protection and survival [64]. Opposite effects are obtained upon activation of mTOR with asparagine, which exacerbates both METH-induced cell death and downregulation of UPS-autophagy merging vacuoles [64]. These findings are in line with studies documenting that inhibition of the mTOR pathway activates UPS-dependent protein degradation besides autophagy [61,62]. In this scenario, mTOR inhibition represents a potential strategy to enhance both autophagy and UPS-dependent protein degradation. The mTOR pathway also regulates the switch between standard and inflammation-inducible UPS subunits forming the immunoproteasome, which is implicated in antigen-peptide processing [104,105,106]. Nonetheless, besides empowering protein degradation, the shuttling of UPS within autophagy vacuoles, which is mediated by p62, may also represent a mechanism aimed at clearing inactive UPS subunits themselves [65,107]. In this functional setting, the merging between UPS and autophagy is defined as “proteaphagy”.

This is in line with studies indicating a reciprocal regulation between the two cell-clearing systems, with UPS behaving as a sentinel in sensing and regulating autophagy, and vice versa [68,108,109,110,111,112,113]. In detail, UPS regulates the duration and amplitude of the autophagy response by controlling the stability of Unc-51 like autophagy activating kinase (ULK1)/Atg1 complex as well as mTOR and transcription factor EB (TFEB) kinases [108,109,110]. ULK1 acts at multiple steps of autophagy initiation and response, in part by phosphorylating autophagy proteins such as Atg13, Beclin 1, and Atg9 [114]. mTOR activation phosphorylates ULK1 to inhibit its kinase activity, thus hampering autophagy initiation [114]. During the early stages of autophagy, UPS mediates the K63-linked polyubiquitination of ULK1 via an E3 ligase (AMBRA1–TRAF6 complex) to maintain its stability, self-association, and kinase activity [108]. Conversely, during prolonged nutrient starvation, UPS targets ULK1 for degradation, thus providing a feedback control of the autophagy response [109].

UPS may control autophagy dynamics also through regulating TFEB turnover, which upon de-phosphorylation and cytoplasm-to-nucleus shuttling, induces the expression of a variety of autophagy-related genes [110,111]. On the one hand, UPS inhibition promotes TFEB accumulation, de-phosphorylation and nuclear translocation, leading to an increased expression of downstream autophagy-related genes including LC3-II, cathepsin D, and LAMP1. Remarkably, despite increasing autophagosome biogenesis, UPS inhibition does not affect autophagy flux [110]. This fits with recent evidence showing that UPS activity, rather than inhibition, promotes autophagy both by fostering the nuclear translocation of TFEB, and remarkably, by degrading mTOR itself, leading to its downregulation and detachment from the lysosomes [111]. In baseline conditions the turnover of the transmembrane autophagy protein Atg9 is also carried out by the UPS, while this is impeded upon autophagy-inducing stimuli such as starvation or rapamycin treatment [112]. Likewise, upon ubiquitination by the ubiquitin-activating enzyme UBA6 and the ubiquitin-conjugating enzyme/ubiquitin ligase BIRC6, LC3-II is targeted for UPS degradation [115,116]. Similar to Atg9, this is occluded following autophagy-stimulating conditions such as nutrient deprivation and protein synthesis inhibition [115,116].

**Figure 1:**
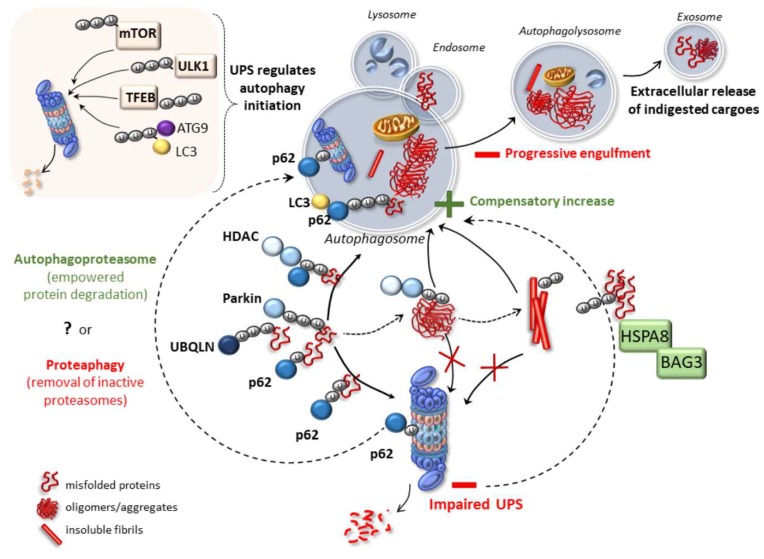
Crosstalk mechanisms between ubiquitin proteasome system (UPS) and autophagy. Ubiquitin tagging is key in sorting misfolded proteins for either UPS- or autophagy-dependent degradation. Several proteins, including UBQLNs, Parkin and SQSTM1/p62, link ubiquitinated misfolded/aggreagted proteins to either UPS or autophagy. Besides misfolded substrates, p62 is key in shuttling the same proteasome to autophagy vacuoles. Functionally, the merging of UPS with autophagy vacuoles may underlie either the formation of a cell-clearing organelle endowed with empowered clearing capacity (the autophagoproteasome), or to the degradation of inactive UPS subunits (proteaphagy). Complementarily, the UPS may control autophagy dynamics by orchestrating the turnover of autophagy-related proteins (LC3 and ATG9) and protein kinases which are implicated in autophagy initiation (mTOR, ULK1 and TFEB). Parkin couples target proteins and mitochondria with dynein motor complexes via the HDAC6 to facilitate their transport towards autophagy compartments. HDAC6 activity is essential for autophagy to compensate for protein degradation when UPS is impaired. This is key when dealing with large oligomers or insoluble fibrils which may occlude the UPS. A UPS impairment may trigger a compensatory increase in autophagy activity. This occurs following the recruitment of HSPA8-BAG3 proteins which reroute protein substrates towards the autophagy pathway. Nonetheless, the accumulation of large, insoluble protein aggregates including oligomers and fibrils, may eventually engulf the autophagy pathway, leading to a prion-like, cell-to-cell propagation of indigested proteins through exosomes release.

These considerations may explain the ambiguous effects that are observed on one cell-clearing system upon inhibition/activation of the other, remarking the importance of autophagy-UPS cooperation in proteostasis. Inhibition of autophagy or UPS alone may be sufficient to impair protein turnover at such an extent that this may affect the efficacy of the other clearing system, thus producing detrimental effects for cell survival, especially within neuronal populations which are mostly susceptible to metabolic alterations [117,118,119,120,121,122,123,124,125]. This is documented, for instance, within dopamine(DA)-containing neurons, which possess a high, inherent susceptibility to DA-related oxidative events fostering post-translational protein alterations [117,118,119,120,121,122,123]. Likewise, in mice bearing a spontaneous mutation in the gene coding for the UPS protein HECT and RLD Domain Containing E3 Ubiquitin Protein Ligase Family Member 1 (HERC1), massive alterations of the autophagy pathway are detected within cerebellar, neocortical, CA3 hippocampal, and spinal cord projection neurons, as evident by the abnormal accumulation of autophagosomes, lysosomes, and altered mitochondria [124,125]. Remarkably, these alterations eventually culminate into neurodegeneration occurring quite selectively within the cerebellar Purkinje cells, likely due to their intense synaptic activity conferring an enhanced inherent susceptibility to UPS and autophagy alterations [124,125]. In some contexts, UPS inhibition may enhance autophagy as an early compensatory response to cope with protein overload in the effort to maintain cell-survival [67,72]. This occurs, for instance, through the HSPA8 and HSPA1A cochaperone BAG3, which is implicated in rerouting UPS substrates to the autophagy pathway [72]. BAG3 induces the sequestration of ubiquitinated substrates into cytoplasmic puncta which colocalize with canonical autophagy markers. BAG3 upregulation, which occurs following UPS inhibition, contributes to the compensatory activation of autophagy and to the degradation of (poly)ubiquitinated proteins. BAG3 binding to the ubiquitinated clients occurs through the BAG domain, in competition with BAG1, which directs ubiquitinated clients to the UPS instead of autophagy. Therefore, the BAG3-BAG1 pathway orchestrates ubiquitinated protein sorting towards either UPS or autophagy, and this may be key to rescue one cell-clearing system when the other is impaired [72]. However, some evidence indicates that autophagy activation following UPS impairment may be only transient, as long-lasting UPS dysfunction impedes mitophagy and decreases the levels of essential autophagy proteins, such as Atg9 and LC3II [70]. In turn, autophagy inhibition leads to the accumulation of ubiquitinated substrates by directly affecting the UPS, either upstream at the level of ubiquitin ligase enzymes, or by decreasing its catalytic activity [68,113]. A summary of the main molecular events implicated in UPS-autophagy cross-talk is provided in Figure 1.

While adding a further level of complexity, these findings indicate that either the synergistic or compensatory interplay occurring between autophagy and UPS needs to be taken into account in experimental approaches modulating either system alone. This may lead to confounding outcomes when assessing the effects of autophagy and UPS separately, while underlining the need for further investigations aimed at finding strategies which can concomitantly rescue defects of autophagy and UPS. In the following paragraph we focus specifically on evidence about the interplay between UPS and autophagy applied to the turnover of specific aggregation-prone proteins.

## 3. Autophagy and Proteasome Interplay in Prion-Like Protein Clearance

### 3.1. Alpha-Synuclein

The understanding of α-syn biology started increasing with the pioneering discovery of a single point mutation in the α-syn gene (*SNCA*), which was associated with autosomal dominant inherited PD [126]. Since then, several *SNCA* alterations contributing to the pathogenesis of PD have been identified. These include point mutations (i.e., A30P, E46K, H50Q, G51D, A53E, A53T) as well as large *SNCA* rearrangements, including gene duplications and triplications [127,128,129]. These gene multiplications produce an excess of normally structured α-syn which leads to severe early-onset familial PD. In fact, *SNCA* multiplications significantly impair life expectancy compared with *SNCA* point mutations, with an exception of the G51D point mutation leading to a devastating parkinsonian-pyramidal disorder overlapping with MSA [130]. Thus, in most cases, normally structured yet overexpressed α-syn may cause brain damage which is worse compared with that induced by mutations leading to an altered protein structure. In these cases, α-syn accumulation is not confined to the brainstem monoamine-containing nuclei which are routinely affected in PD [131,132]. In fact, it may extend further to involve a variety of brain areas and even the spinal cord, possibly through a prion-like propagation route [131,133,134]. For instance, α-syn spreading to cortical regions is associated with Dementia with Lewy Bodies (DLB), while α-syn aggregates occur in both neurons and oligodendrocytes in MSA, thus contributing to a massive de-myelinization within affected brain areas [135,136]. Besides classic synucleinopathies, α-syn aggregates may be detected in a variety of disorders including Progressive Supranuclear Palsy (PSP), HD, AD, FTD, ALS, and brain ischemia [23,24,25,26,136,137]. A toxic gain of function is supposed to underlie α-syn overexpression, as confirmed by experimental models of parkinsonism including administration of METH [103,119,120,138], MPTP [139,140,141], and 6-OHDA [142,143]. These neurotoxins impair autophagy and UPS, suggesting that impaired α-syn clearance represents a common mechanism in a variety of neurological disorders.

While experimental evidence on the role of UPS in metabolizing α-syn dates back to the early 2000s [52,117,118,119,140,144,145,146,147] the implication of autophagy was evident a few years later, and fully confirmed in recent in vivo studies on *ATG7*-depleted DA neurons [121,122,123]. These studies indicate that both UPS and autophagy are seminal for the survival of DA neurons, since inhibition of either pathway leads to parkinsonism featuring a massive loss of midbrain DA neurons and α-syn accumulation within ubiquitin-positive Lewy body-like inclusions in surviving neurons [119,121,122,140]. Intriguingly, α-syn accumulation following UPS inhibition in vivo may also occur in the absence of ubiquitylation, and unmodified α-syn can be directly degraded by the P20S through an ubiquitin-independent mechanism in vitro [148].

However, the contribution of UPS compared with autophagy remains a matter of investigation. α-Syn degradative pathways were concomitantly investigated in a PC12 cell model expressing exogenous human wild-type (WT) or mutated (A30P, or A53T) α-syn [53]. By using a panel of inhibitors/stimulators of autophagy and UPS it was found that α-syn is degraded by autophagy besides the UPS. A role of autophagy is supported by evidence on the ultrastructural occurrence of α-syn within autophagy-like vesicles. Rapamycin, an autophagy inducer, remarkably increases α-syn clearance, suggesting that autophagy represents the major degradation pathway for synuclein [53].

Beyond point mutations conferring a toxic gain of function, α-syn dosage is an important modulator of its cellular toxicity. In an inducible PC12 cell model expressing WT or mutated A30P α-syn, the dosage-dependent effects of α-syn were explored in relation with UPS and autophagy clearing capacity [149]. At low expression levels, neither WT nor mutated (A30P) α-syn aggregate or produce toxicity. Remarkably, they also protect against hydrogen peroxide (H_2_O_2_)-induced oxidative stress. However, when both WT and A30P α-syn are overexpressed, such an antioxidant function is no longer detectable. It is noteworthy that no α-syn aggregation and cell death occur until autophagy or UPS inhibitors are administered [149]. Either autophagy or UPS inhibitors enhance A30P α-syn toxicity. On the other hand, autophagy but not UPS inhibition induces WT α-syn accumulation along with the occurrence of oligomer-positive aggregates and cell toxicity. At a first glance, this would suggest that both autophagy and UPS are necessary to clear mutated α-syn, while autophagy is the preferential route for WT and oligomeric α-syn degradation [149]. However, this may be a rather simplistic conclusion if one considers that autophagy inhibitors also affect UPS activity, and in turn, UPS inhibitors may produce a compensatory increase in autophagy activity. In fact, as reported by in vivo studies, the UPS is the main degradation pathway for α-syn in baseline conditions, while an increased α-syn burden recruits the autophagy pathway. UPS alterations occur early in α-syn transgenic mice, and UPS decline may lead to an upregulation of the autophagy pathway to cope with α-syn overload [150].

Such an issue was addressed by further studies combining autophagy and UPS inhibitors. In detail, when both the UPS and autophagy are inhibited with MG132 or epoxomicin and 3-MA, respectively, a dramatic increase in both WT and mutant A53T α-syn levels is detected when compared with treatment using either single agent [68,80]. This goes along with a decrease in cell viability in both WT and mutant α-syn expressing cells [68,80]. Intriguingly, beyond epoxomicin, 3-MA also decreases chymotrypsin-like UPS activity while increasing E2 ligase expression, which indicates a complex interdependency between the two systems. When the autophagy inducers rapamycin or trehalose are administered to epoxomicin- or MG132-treated cells, α-syn expression and accumulation as well as cell loss are remarkably prevented [68,80].

These findings are in line with recent in vitro studies showing that both UPS and autophagy degrade both WT- and E46K mutant α-syn, though this latter is turned over more slowly compared with WT isoform [151]. Thus, both autophagy and UPS are needed to counteract the increased vulnerability to apoptosis associated with the overexpression of mutant when compared with WT α-syn [151]. These results suggest a coordinated, potentially synergistic activity between autophagy and UPS to prevent α-syn accumulation and toxicity. Still, when UPS is impaired, autophagy activation may cope with α-syn clearance to rescue cell survival [80]. This is important since misfolded α-syn overexpression early impairs UPS function preceding the onset of behavioural deficits and DA neuron degeneration, and this is associated with selective accumulation of neurotoxic α-syn phosphorylated at the serine 129 residue [79].

A further level of complexity emerges when considering that a number of additional, yet probably unexplored, common molecular pathways besides mTOR are implicated in autophagy and UPS cross-talk. This is the case of USP9X, which is implicated in α-syn deubiquitination and it is markedly decreased in α-synucleinopathies [152]. Remarkably, de-ubiquitination of α-syn by USP9X determines the partition of α-syn between the UPS and autophagy pathways. While mono-ubiquitinated α-syn is degraded by the UPS, de-ubiquitination of α-syn favors its degradation by autophagy [152]. This suggests that modulation of USP9X represents a strategy for rerouting α-syn to the autophagy pathway when UPS is overwhelmed or impaired, at least at an early stage when autophagy flux is still not consistently affected. In this context, it was recently shown that downregulating two enzymes which are involved in ubiquitin conjugation and substrates targeting for UPS, namely UBA6 or BIRC6, decreases α-syn aggregates in rat hippocampal neurons. This occurs via autophagy rescue due to limited UPS-dependent degradation of LC3II [115,116].

Again, F-box only protein 7 (FBXO7), a substrate recognition component of an E3 ubiquitin-protein ligase complex associated with both UPS and autophagy activity [153,154], may be a potential target in synucleinopathies as well. In fact, FBXO7 is detected within α-syn-positive inclusions (Lewy bodies and neurites, and glial cytoplasmic inclusions), where it co-localizes with α-syn in both PD and MSA human cases [155]. Besides associating with α-syn aggregation, FBXO7 may itself behave as a prionoid. In fact, either overexpression of WT *FBXO7* or mutated *FBXO7* may lead to FBXO7 aggregation which is associated with impaired mitophagy and DA neuron degeneration in animal models [156]. At baseline, FBXO7 directly interacts with Parkin to promote mitophagy. A loss of FBXO7 function impairs the translocation of Parkin to mitochondria, as well as ubiquitination of mitofusin 1 and mitophagy, while rescuing FBXO7 function reverses these effects [154].

Besides interacting with Parkin, FBXO7 also targets (i) glycogen synthase kinase 3β (Gsk3β), which is involved in α-syn phosphorylation and autophagy, and (ii) the translocase of outer mitochondrial membrane 20 (Tomm20), which upon ubiquitination promotes mitophagy [157]. These findings indicate that FBXO7 intermingles with UPS and autophagy to guarantee protein and mitochondrial homeostasis, while its alterations may be bound to impaired targeting and clearance of UPS and autophagy substrates. Targeting FBXO7 alterations rescues UPS and autophagy, which may be beneficial for α-syn aggregation as well, though it still remains to be investigated whether and how UPS and autophagy modulators may influence FBXO7 dynamics and function.

### 3.2. Amyloid Beta and tau

Amyloid-β (Aβ) deposits in extracellular senile and neuritic plaques, along with hyperphosphorylated tau proteins in intracellular neurofibrillary tangles (NFT), are hallmarks of AD. Aβ is a heterogeneous mixture of small peptides, 37–43 amino acids in length, that are generated by sequential cleavage of the amyloid precursor protein (APP) by β- and γ-secretase [18,158]. Besides AD, Parkinsonian Lewy pathology is associated with widespread Aβ pathology, indicating a link between Aβ accumulation and PD [159]. Accumulation of AD-like proteins, including aggregated Aβ-precursor protein 751 (AβPP751), Aβ, and phosphorylated tau also occurs in muscular disorders such as sporadic inclusion-body myositis [160]. Likewise, deposition of abnormal microtubule-associated tau protein within the cerebral tissue characterizes a number of disorders besides AD, including progressive supranuclear palsy, traumatic encephalopathy and FTD [161].

As thoroughly reviewed, both autophagy and UPS mediate Aβ and tau degradation, while their failure in AD experimental models and humans is associated with Aβ and Tau accumulation and toxicity [54,162]. Impaired autophagy and UPS also enhance Aβ42-induced learning and memory deficits in AD animal models [163]. Both in vitro and in vivo, autophagy and UPS mainly clear oligomeric Aβ42, while monomeric Aβ42 species are removed preferentially via the endo-lysosomal system [163]. In human SH-SY5Y neuroblastoma cells transfected with either WT or mutant AβPP gene, the overexpression of the AβPP mutant isoform correlates with an increase in oxidative stress along with a marked inhibition of UPS and at a lesser extent, of the autophagy flux [73]. In fact, cells try to promote autophagy in a HDAC6-dependent manner as a compensatory response. Nonetheless, treatment with Aβ42 oligomers eventually occludes USP and autophagy activity up to neuronal degeneration, suggesting the existence of an Aβ42 threshold level beyond which UPS-dependent proteolysis becomes definitely dysfunctional [73].

The E3 ubiquitin ligase Parkin, which is implicated in protein ubiquitination and degradation as well as autophagy/mitophagy stimulation, was recently shown to be reduced in AD besides PD [164]. In AD models, Parkin interacts with misfolded Aβ targeting it for UPS- and autophagy-dependent clearance. In vitro, WT Parkin decreases baseline levels of intracellular Aβ, while such an effect vanishes following UPS inhibition. In turn, intracellular Aβ accumulation impairs UPS activity while decreasing cell viability [164]. Accordingly, Parkin reverses all these effects, while Parkin knock-down exacerbates accumulation of Aβ along with UPS failure [164]. Similarly, in transgenic AD mice models, lentiviral parkin expression ubiquitinates Aβ to reduce its intracellular levels while preventing plaque deposition, and this is associated with induction of beclin-dependent autophagy [165]. Thus, parkin reduces Aβ levels by enhancing both UPS- and autophagy-dependent clearance of Aβ. Parkin-mediated clearance of ubiquitinated Aβ may act in parallel with autophagy to clear various debris, including defective mitochondria.

In the brains of both AD patients and transgenic-mouse models, phosphorylation of neuronal Aβ precursor protein (AβPP) on Thr668 is considered to be detrimental since it increases cytotoxic Aβ and induces tau phosphorylation. Activated GSK3β is involved in phosphorylation of both Aβ and tau [166,167,168,169]. In line with this, lithium, a GSK3β inhibitor and autophagy inducer, confers neuroprotection by reducing the burden of Aβ plaques as well as p-Aβ and p-tau in a variety of AD animal models [166,167,168,169]. This is recapitulated in AβPP-overexpressing cultured human muscle fibers [160]. Remarkably, in human muscle cultures it was shown that UPS inhibition significantly increases GSK3β activity and AβPP phosphorylation; conversely, treatment with lithium produces an increase in UPS activity which goes along with decreased levels of phosphorylated-AβPP, as well as reduced amount of total AβPP and Aβ oligomers [160]. These findings add to the evidence linking lithium-induced inhibition of GSK3β activity with neuroprotection and autophagy induction [59,60,170], showing that GSK3β inhibition may empower UPS besides autophagy to prevent Aβ accumulation.

Supporting the role of a concomitant impairment of autophagy and UPS in AD, recent studies show that BACE2, a β-site APP-cleaving enzyme which contributes to Aβ generation, is degraded by both the UPS and autophagy in neuronal and non-neuronal cells [171]. This suggests that BACE2 dysregulation and Aβ accumulation might be due to autophagy and UPS impairment in AD.

However, one should consider that an alternative stream of interpretation exists, suggesting that autophagy activation following UPS failure may foster accumulation and extracellular spreading of Aβ [69]. For instance, in SH-SY5Y neuroblastoma cells transfected with AβPP, UPS inhibition leads to Aβ accumulation within autophagosomes and lysosomes [69]. This is associated with a reduced degradation of the C-terminal fragment (C99) of AβPP by the UPS, making it available for γ-secretase cleavage, thus increasing both intracellular and secreted Aβ levels. Intriguingly, autophagy blockade following UPS inhibition reverses such an effect while further increasing the C99 to AβPP ratio. Supporting autophagy involvement in Aβ generation, UPS inhibition produces a reduction in cellular viability, while autophagy inhibition ameliorates rather than exacerbates such an effect [69]. These findings suggest that UPS failure may lead to accumulation of Aβ within autophagy compartments fostering increased generation and secretion of Aβ [69]. One may argue that this is due to an engulfment of autophagy structures or an impairment of autophagy flux. However, an increase in cellular viability is detected following concomitant inhibition of autophagy and UPS compared with UPS inhibition alone, suggesting that autophagy may be recruited as an unsuccessful compensatory mechanism which instead of being protective, exacerbates Aβ secretion.

The implication of both autophagy and UPS in clearing pathological tau is increasingly recognized. However, their specific contributions to normal and pathological tau clearance, and their impairment at specific stages of AD pathology, remain unclear. A line of evidence suggests that defective autophagy might primarily contribute to the accumulation of tau in neurons [165]. By using both WT and phosphomimic mutant forms of tau, its phosphorylation effects were investigated concerning the rate of axonal transport and degradation by the UPS and autophagy [172]. Phosphomimic tau shows reduced binding to microtubules and exhibits an increased number of motile tau particles in axons compared with WT tau. In primary cortical neurons, both exogenously expressed isoforms of tau, namely phosphomimic and WT tau, are autophagy substrates, though endogenous tau is not. In fact, autophagy inhibition in neurons leads to a 3-fold accumulation of phosphomimic tau compared with WT tau, while endogenous tau is unaffected [172]. In autophagy-deficient mouse embryonic fibroblasts, but not in neurons, UPS-dependent degradation of phosphomutant tau is also reduced compared with WT tau. In the absence of autophagy, degradation of tau by the UPS in neurons is minimal. These findings suggest that despite both autophagy and UPS being involved in tau degradation, autophagy may be the primary route for clearing phosphorylated tau in neurons [172].

Independently of tau phosphorylation, the increased expression of the DNA Polymerase Delta Interacting Protein 2 (*POLDIP2*) following either ectopic manipulation or exposure to Aβ, may enhance the formation of tau aggregates by impairing both autophagy and UPS [173]. In a drosophila model of human tauopathy, knockdown of the drosophila *POLDIP2* homolog attenuates tau overexpression-related phenotypes and it increases flies’ lifespan while rescuing autophagy and UPS activities [173].

Recent studies support a hypothesis according to which autophagy may be perturbed in later AD stages compared with UPS, since UPS impairment leads to an early compensatory recruitment of autophagy coping with tau clearance [174]. For instance, the negative regulator of ubiquitin-like protein 1 (NUB1) is an UBL/UBA protein fostering the targeting of protein substrates for UPS degradation [174]. Remarkably, NUB1 reduces tau phosphorylation and aggregation following UPS inhibition, leading to a switch towards the autophagy pathway. In detail, during UPS inhibition, NUB1 interacts specifically with p62 via the UBA domain to enhance its levels while increasing autophagosomes and the recruitment of lysosomes to aggresomes. In line with this, NUB1 moves to cytosolic inclusions containing pathological forms of tau, as well as LAMP1 and p62 in the hippocampal neurons of tauopathy-bearing mice. Thus, NUB1 contributes to tau removal by regulating the autophagy pathway upon UPS impairment [174].

### 3.3. Huntingtin

Mutations in *Huntingtin* cause autosomal dominant HD. Such mutations consist of expansions in the polyglutamine domain of the protein surpassing the threshold of 36 glutamines. The length of mutant polyQ expansion strongly correlates with aggregation rate, in an inverse manner to disease age of onset [175].

UPS and autophagy were concomitantly investigated concerning protein clearance dynamics by using exon 1 of the *Huntingtin* gene with expanded polyglutamine repeats and enhanced green fluorescent protein tagged to 19 alanines as models for aggregate-prone proteins [81]. Autophagy is clearly involved in the degradation of these proteins, since they accumulate when cells are treated with different autophagy inhibitors acting at distinct autophagy stages. Conversely, rapamycin promotes the clearance of huntingtin aggregates while reducing cell death associated with the polyglutamine and polyalanine protein expansions [81]. When both lactacystin and the specific UPS inhibitor epoxomicin are administered, an increase in soluble protein levels of the polyglutamine constructs is detected, suggesting that soluble huntingtin isoforms are also a substrate of UPS. However, while polyglutamine-protein aggregation is enhanced by lactacystin, such an aggregation is paradoxically reduced by epoxomicin, suggesting that unknown proteins/pathways induced by epoxomicin may regulate polyglutamine-huntingtin aggregation. This is likely to occur by acting at the level of autophagy [81].

Indeed, very little is known about the effects of an enhanced UPS activity on the autophagy flux and consequent rate of protein degradation. In the context of huntingtin metabolism, recent studies unraveled a role for the deubiquitinase ubiquitin specific peptidase 14 (USP14) as a common denominator mediating a compensatory negative feedback between the two major proteolytic pathways [176,177,178]. On the one hand, UPS activation achieved through USP14 inhibition facilitates the clearance of tau and reduces the amount of its oligomeric forms. On the other hand, it increases the formation of inclusion bodies containing huntingtin with long polyglutamine repeats. This is likely due to the fact that an upregulation of UPS activity, through the inhibition of USP14, significantly impairs cellular autophagy flux, especially at the autophagosome-lysosome fusion step [176,177]. This may occlude the degradation of large, non-proteasomal substrates. Potential mechanistic insights on the role of UPS14 in the coordinated activities between UPS and autophagy were also provided [178]. In detail, UPS inhibition disrupts the association of USP14 with the P19S regulatory particle of the UPS, while fostering its interaction with either the autophagy protein GABARAP or the heat-shock-related co-chaperonin HSC70 [178]. Striatal neurons expressing mutant huntingtin possess reduced USP14 levels, while USP14 overexpression increases GABARAP positive autophagosomes in striatal neurons [178]. This suggests that in the presence of mutant huntingtin, low USP14 levels may contribute to UPS activity prevailing over the autophagy pathway, while co-chaperonins and the autophagy pathway may be recruited at later stages when large HT aggregates accumulate in the cell. Modulating USP14 levels may be a potential strategy switching to the autophagy pathway when UPS is overwhelmed. These results present novel mechanistic insights into the reciprocal communication between the UPS and autophagy. At the same time this suggests that strategies upregulating either the UPS or autophagy hold great potential, albeit possessing caveats which originate from the intrinsic feedback regulation occurring between the two degradation systems [177].

### 3.4. TDP-43

TAR DNA-binding protein 43 (TDP-43) is a main constituent of cytoplasmic aggregates in neuronal and glial cells in ALS and FTD. Treatment with either MG132 UPS inhibitor, or 3-MA autophagy inhibitor, increases the protein levels of TDP-43 and its truncated pathological form TDP-25 consisting of the “prion-like” domain [179]. Conversely, when the autophagy inducer trehalose is administered, both TDP-25 and TDP-43 levels are decreased. Nonetheless, TDP-25 levels are more affected compared with TDP-43 under the effect of inhibitors or stimulators. These data suggest that TDP-43 and TDP-25 are degraded by both UPS and autophagy, with TDP-25 being more prone to autophagy regulation [179]. Scotter et al. showed that soluble TDP-43 is degraded primarily by UPS, while the clearance of aggregated TDP-43 requires autophagy [77]. Large cellular aggregates, which recapitulate many of the pathological features occurring in patients, are reversible when both the UPS and autophagy are functional, though autophagy is mostly implicated in the clearance of oligomeric TDP-43 isoforms. Thus, adding to an age-related decline in cell-clearing activity, a second hit in either the UPS or the autophagy pathway may occur, driving the accumulation of TDP-43 in ALS and FTD [77].

Autophagy-mediated degradation of aggregation-prone proteins relies on an active retrograde transport, mediated by dynein complexes and specific chaperones including the HSPB8-BAG3-HSPA8 axis [180]. Intriguingly, in ALS cell models, inhibition of dynein-mediated retrograde transport, which impairs the targeting to autophagy of misfolded species, does not increase the aggregation of truncated TDP-43 or mutant SOD1. In fact, dynein inhibition correlates with an increased clearance of truncated TDP-43 and mutant SOD1 by the UPS, which occurs as a compensatory response due to the upregulation of the HSPA8 co-chaperone BAG1. Thus, when the misfolded proteins cannot be efficiently transported toward the perinuclear region of the cells to be degraded by autophagy, BAG1 is recruited as a compensatory mechanism to target the HSPA8-bound cargo to the UPS in a dynein-independent manner [180].

Recent studies suggest that different TDP species along with intrinsic cell properties may differently affect protein accumulation and clearance rate [71]. This was assessed in a comparative study in immortalized motoneuron-like cells and stabilized myoblasts. Remarkably, in both cell types, both TDP-25 and TDP-43 are cleared by UPS. However, TDP-25, which is the prevailing species in motor neurons, specifically impairs the autophagy pathway. This seems to fit well with previous findings showing a prevailing role for UPS in the clearance of TDP-43, which occurs following BAG1 recruitment. In line with this, TDP-25 accumulation in both cells decreases upon either BAG1 overexpression, which routes TDP-25 fragment to UPS. Noteworthy, the reversal of TDP-25 accumulation is reproduced following BAG3 overexpression as well, which routes TDP-25 fragment to autophagy instead. In this context BAG3/BAG1 modulation appears as a novel strategy to rescue one cell-clearing system when the other is impaired [71].

### 3.5. FUS

Besides TDP-43, fused in sarcoma (FUS) is a main constituent of cytoplasmic aggregates occurring in familial and sporadic ALS. In cultured murine-derived neurons, motor neurons, astrocytes and oligodendrocytes, infection with recombinant adenovirus vectors encoding WT and mutant FUS leads to intracellular FUS aggregates formation, which is exacerbated upon co-infection with short hairpin RNAs (shRNAs) for the 26S proteasome regulatory subunit 1 *PSMC1* and the autophagy gene *ATG5* [181]. This is recapitulated in vivo, where ablation of these UPS and autophagy genes produces a dramatic increase in the expression of FUS within motor neurons. These very same effects are observed on TDP-43 aggregation [181]. These findings suggest that a concomitant impairment of UPS and autophagy pathways may accelerate formation of FUS-positive aggregates in ALS [181].

It is now quite established that nucleus-to-cytoplasm mislocalization of FUS, similar to TDP-43, may be key to fostering its intracellular aggregation and toxicity. In this context, autophagy activation reduces cytoplasmic FUS aggregates while rescuing motor function in vivo [182]. Nonetheless, evidence exists indicating that UPS also plays a key role, especially in the nuclear metabolism of FUS. In a mouse model of Down syndrome (Ts65Dn mice), Purkinje cells undergo degeneration which is linked with UPS inhibition and FUS accumulation [183]. In fact, UPS chymotrypsin-like proteolytic activity is reduced by 35% in the cerebellum of Ts65Dn mice compared with WT animals. This is associated with a massive increase of intra-nuclear ubiquitinated FUS within Purkinje cells and large neurons of cerebellar nuclei.

Recent studies unraveled a role of Mask, an Ankyrin-repeat and KH-domain containing protein, in promoting autophagy-dependent FUS clearance when UPS is impaired [184]. In Drosophila models of ASL bearing an eye-specific expression of human pathogenic FUS, loss of Mask enhances eye degeneration while Mask gain of function mitigates such an effect. By using fly larval muscle, it was found that Mask modulates the amount of K48- and K63-ubiquitinated proteins by regulating autophagy-lysosome-mediated degradation [184]. Indeed, upregulation of Mask compensates the partial loss of UPS function. In detail, by promoting the expression levels of the proton-pumping vacuolar (V)-type ATPase, Mask enhances lysosomal function and autophagy flux [184]. These findings highlight the importance of lysosome acidification besides autophagosomes’ dynamics when the UPS is impaired. Further studies employing modulators of both pathways are needed to disclose any potential interplay mechanisms applied to FUS protein metabolism specifically.

### 3.6. SOD1

In spinal cord motor neurons from transgenic G93A-SOD1 mice, mutant SOD1 accumulates along with the small heat shock protein HspB8 [185]. The latter decreases mutant SOD1 aggregation while increasing its solubility and clearance, without affecting WT SOD1 turnover. Remarkably, HspB8 acts on mutant SOD1 even when the UPS activity is specifically blocked, indicating a role for compensatory autophagy activation. In fact, the pharmacological blockage of autophagy leads to a dramatic increase of mutant SOD1 aggregates. During autophagy flux blockage, mutant SOD1 interacts with the HSPB8/BAG3 complex, which is known to activate autophagy-mediated removal of misfolded proteins, suggesting that HSPB8 increases mutant SOD1 clearance via autophagy [185]. Similar to what has been reported for TDP-43, these results indicate that the pharmacological modulation of HSPB8/BAG3 expression in motor neurons may have important implications to unravel the molecular mechanisms bound with cell-clearing systems alterations in both familiar and sporadic ALS.

Similar to what has been reported for TDP-43, muscle cells besides motor neurons might be a target of SOD1 toxicity [186,187]. In line with this, muscle-restricted expression of the fALS protein mutant SOD1 induces muscle atrophy and motor neuron death. Intriguingly, the clearance of misfolded mutant SOD1 appears to be much more efficient in muscle cells compared with motor neurons [186,187]. In fact, mutant SOD1 forms aggregate and impair UPS only in motor neurons, while in muscle cells it remains soluble even when UPS is inhibited. The higher mutant SOD1 clearance in muscle cells correlates with a more efficient UPS activity, which goes along with a robust autophagy activation. Therefore, autophagy and UPS seem to better manage misfolded SOD1 species in muscle cells compared with motor neurons [186,187]. In line with this, muscle ALS models possess much higher chymotrypsin UPS activity and autophagy power compared with motor neuron ALS models.

## 4. Cell-Clearing Systems and Prion-Like Protein Propagation

The intracytoplasmic deposition of protein aggregates associated with a failure in UPS and autophagy pathways may affect the neighboring cells through cell-to-cell transmission of these aggregates. Such an increased release of prion-like proteins in the extracellular space could promote their propagation to different CNS areas, ultimately spreading pathology and sustaining disease progression. Most studies are focused on α-syn, though more evidence is available suggesting a leading role for autophagy rather than UPS alterations in pathological α-syn spreading. In fact, pharmacological and genetic inhibition of autophagy increases α-syn exocytosis and transcellular propagation in mixed cultures [48]. Such an increase in protein transmission is associated with apoptotic cell death in the recipient cells [48]. In mice models, bafilomycin-induced autophagy inhibition reduces intracellular α-syn aggregation and it also increases the secretion of smaller oligomers that exacerbate microenvironmental responses including α-syn uptake, inflammation, and cellular damage [49]. Low-aggregated α-syn is predominantly released by exosomes and endosome-associated pathways, while high-aggregated α-syn is secreted by membrane shedding. These findings suggest that the toxic role of α-syn is mostly related to its extracellular species rather than intracellular protein aggregates. Thus, rather than limiting intracellular α-syn aggregation, α-syn clearance is key to preventing extracellular α-syn release which occurs mostly via autophagy-related secretion of extracellular vesicles (EVs) [49]. In fact, EVs are generated in the multivesicular body compartment to be either released upon fusion with the plasma membrane, or cleared via the autophagy pathway. In line with this, inhibiting autophagy increases extracellular shuttling of multivesicular body contents to enhance α-syn extracellular release and cell-to-cell transfer [188]. Indeed, autophagy inhibition remarkably increases the ratio of extra- to intracellular α-syn while upregulating α-syn association with EVs in neuronal cells [188]. This is confirmed by ultrastructural and biochemical characterization documenting a widespread, fused multivesicular body-autophagosome compartment along with the presence of autophagosome-related proteins, such as LC3-II and p62 [188]. These features are also identified in EVs from the cerebrospinal fluid (CSF) of DLB patients. Following mice intracortical injection with α-syn-positive EVs from DLB CSF, human α-syn co-localizes with endosome and neuronal markers [188]. Therefore, autophagy inhibition increases α-syn in neuronal EVs, and this may correlate with α-syn-EVs transfer from cell to cell, according to a prion-like propagation. Nonetheless, it is conceivable that a dysfunction in either clearance pathway, or a combination thereof, may be involved in the extracellular release of undigested α-syn, fostering prion-like pathology.

In fact, a few studies also report a direct involvement of UPS in cell-to-cell protein transmission. UPS failure may foster accumulation and extracellular spreading of Aβ, though this is paradoxically associated with autophagy activation [69]. In fact, in neuroblastoma cells transfected with AβPP, UPS inhibition leads to intracellular Aβ accumulation within autophagosomes and lysosomes, which in turn, is associated with a subsequent increase in secreted Aβ levels. Autophagy blockade reverses such an effect, suggesting that compensatory autophagy activation following UPS inhibition may promote Aβ extracellular secretion [69].

In a cellular model of preformed tau fibrils, it was shown that tau aggregates can be gradually cleared only when soluble tau expression is suppressed [189]. This clearance is mostly mediated by autophagy, though both the UPS and autophagy pathway are deficient when handling large tau aggregates, which occur once soluble tau expression is turned on again. Remarkably, these tau aggregates are dynamic structures constantly undergoing “fission” and “fusion,” which facilitate stable propagation of tau pathology in dividing cells. This suggests that indigested, large tau aggregates which are mostly autophagy substrates, may promote cell-to-cell spreading, propagating tau pathology [189].

Again, in exogenous TDP-43-expressing cells, UPS inhibition induces TDP-43 aggregates containing phosphorylated TDP-43 [190]. These aggregates remain insoluble in culture media, consisting of sarkosyl-insoluble granular materials, and remarkably, they are released into neighboring neuronal cells, suggesting a cell-to-cell propagation [190].

Despite these pieces of evidence, further studies are needed to investigate the specific role of UPS, as well as UPS-autophagy interplay in prion-like protein spreading.

## 5. Protein Glycation and Cell-Clearing Systems Alterations Bridging Cell-to-Cell Propagation and Neuro-Inflammation

Post-translational protein modifications play a key role in modifying protein structure and conformation, thus contributing to protein misfolding, aggregation and spreading. Besides genetic mutations, the cellular environment may lead to spontaneous protein misfolding. This is based on conformational changes where α-helix protein domains are converted into β-sheet structures. For instance, oxidative compounds may promote protein misfolding and subsequent aggregation through the oxidation of SH groups into S-S disulphide bonds [191]. Furthermore, specific sugar residues may bind to proteins leading to a spontaneous reaction to form a Shiff’s base, fostering protein glycation [192,193]. Glycation represents one of the post-translational modifications of prion-like proteins, including α-syn, Aβ, tau, huntingtin and SOD-1 [193,194,195,196,197,198,199]. Glycation eventually leads to the formation of advanced glycation end products (AGEs) which may either derive from a cascade of spontaneous reactions, or also directly from the binding of the proteins to methylglyoxal, glyoxal and 3-deoxyglucosone compounds.

Depending on the protein, glycation can both promote amyloid aggregation or induce the formation of oligomeric species being stabilized by covalent AGE-derived cross-links [200]. Glycation can affect structural and physicochemical features of amyloid oligomers as well as their interaction to the cell membrane, and subsequently influence or induce cell toxicity. Based on the fact that AGE themselves are insoluble molecules with cytotoxic effects, they are not digested by autophagy and UPS [196]. In fact, formation of AGE-derived cross-linked proteins may inhibit the UPS due to their bulky structure, leading to an increase in oxidized and damaged proteins, which, in turn, may promote protein aggregation and further AGEs formation, engulfing the autophagy compartments [193,201]. Thus, AGEs make misfolded protein aggregates irreversible and protease-resistant, likely increasing their propensity to spread from cell to cell [193,195]. In this scenario, undigested AGEs and AGE-modified proteins, being substrates of the autophagy-lysosomal system [202], can be released extracellularly upon merging with the plasma membrane.

**Figure 2:**
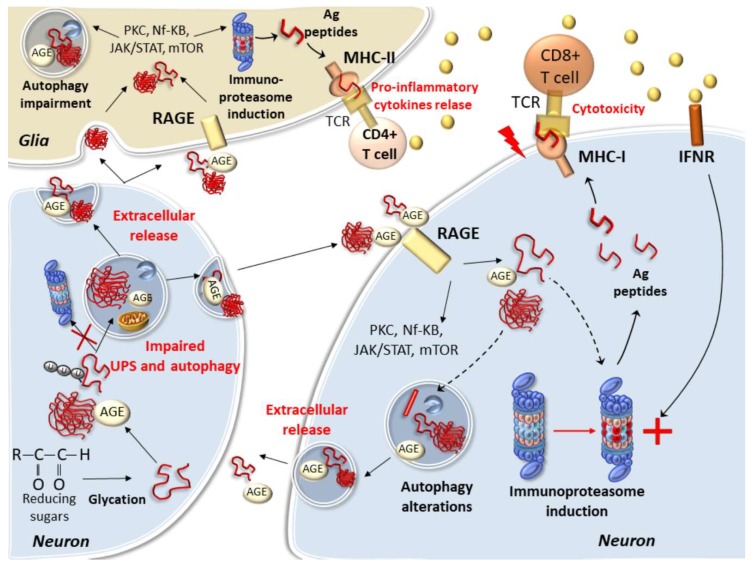
Autophagy and UPS alterations bridging altered proteostasis with neuroinflammation, the paradigm of protein glycation. Within neurons, glycation leads to the formation of advanced glycation end products (AGEs) which structurally modify proteins while promoting the formation of oligomer species. These in turn may impair the UPS while engulfing the autophagy pathway. Thus, indigested AGE-modified proteins may be released extracellularly upon merging of autophagy compartments with the plasma membrane. In neighboring cells, including neurons and glia, the presence of RAGEs allows binding and entering of prionoids. While spreading prionoids in neighboring cells, the binding of AGEs with RAGEs triggers a variety of transduction mechanisms which bridge alterations in cell-clearing mechanisms with inflammatory events. In fact, the binding of AGEs with RAGEs triggers the activation of PKC, NF-kB, JAK2/STAT1 and mTOR pathways, which promote a vicious cycle of inflammatory reactions while leading to the replacement of standard UPS subunits with immune-related ones, called immunoproteasomes. Besides fostering the replacement of standard- with immune-proteasome subunits, these pathways may also impair the autophagy machinery. This may contribute to further promote the extracellular release of indigested AGE-modified proteins. At the same time, the immunoproteasome triggers a variety of inflammatory and immune reactions by cleaving misfolded proteins specifically within immunogenic sites. This leads to the production of Ag peptides that are recognized by MHC-I and MHC-II in neurons and glia, respectively. The cognate Ag/MHC-I complex exposed on the neuronal plasma membrane induces proliferation of CD8+ T-cells while triggering CTL-mediated neuronal death. At the same time, Ag-peptides derived from the degradation of misfolded proteins which are phagocytosed by or routed via RAGEs within glial cells can be presented via MHC-II molecules for re-activation of CD4+ T cells. In this context, the immunoproteasome may cross-process Ags which bind on MHC-II molecules to prime CD4+ Th lymphocytes and fuel production of pro-inflammatory cytokines in the brain milieu. Cytokines, in turn, may further promote immunoproteasome induction within neurons through interferon receptors, leading to a vicious cycle of events bridging alterations in protein degradation with prion-like spreading and neuroinflammation.

In neighboring cells, the presence of specific AGE receptors, termed RAGEs, allow binding and entering of prionoids. Thus, AGE-modified misfolded proteins can either aggregate and persist in the donor cell or move out to spread the disease within recipient cells expressing RAGEs. These include brain endothelial cells, microglia, astrocytes and neurons [203,204]. RAGEs possess three domains which belong the immunoglobulin family, one extracellular (variable) V-type domain, which binds AGEs and β-sheet chains, and two (constant) C-types which anchor the receptor to the cell membrane. Remarkably, while spreading prionoids in neighboring cells, the binding of AGEs with RAGEs triggers a variety of transduction mechanisms which may bridge alterations in cell-clearing mechanisms with apoptotic and inflammatory events (Figure 2) [205]. In detail, RAGEs trigger activation of PKC, NF-kB and JAK2/STAT1 pathways, which promote a vicious cycle of inflammatory and oxidative reactions while leading to the replacement of standard UPS subunits with immune-related ones, called immunoproteasomes [205].

The immunoproteasome is an alternative UPS isoform implicated in antigen (Ag) processing and presentation, which operates constitutively in immune cells while being induced by pro-inflammatory and oxidative stimuli in almost any other cell type [104,105,106,205,206,207,208,209,210,211]. In fact, the immunoproteasome is significantly upregulated in reactive astrocytes, microglia, and neurons in both patients and experimental models of neurodegeneration [104,105,106,205,206,207,208,209,210,211,212,213]. Compared with standard proteasome, the immunoproteasome owns enhanced chymotrypsin-like activity and structural peculiarities which enable fast and efficient processing of endogenous, microbial- and misfold/aggregation-prone-proteins [104,206,207,208]. In fact, the immunoproteasome is recruited as a compensatory, protective attempt to cope with protein overload and cellular stress when standard UPS is impaired, thus contributing to the regulation of misfolded protein-driven innate immune responses [105,207,208,211]. Immune adaptation of the UPS is a tightly regulated and transient response, as cells rapidly need to switch back to standard UPS once immunoproteasome function is no longer required [214]. Nonetheless, under persistent inflammatory and oxidative stimuli which occur during protein aggregation and spreading, the immunoproteasome may abnormally persist within glial and neuronal cells, fueling a vicious cycle of inflammatory and immune reactions in the CNS [104,211].

In detail, the immunoproteasome cleaves misfold-prone proteins specifically within immunogenic sites, to trigger inflammatory and immune reactions through Ag presentation by MHC-I molecules and subsequent activation of CD8+ T lymphocytes [208,210]. Recent studies show that besides microglia and astrocytes, neurons express MHC-I molecules as well, behaving as competent antigen presenting cells [210,213,215]. Low levels of MHC-I are also detected in baseline conditions in the absence of cytokine stimulation in both glial and neuronal cells; however, MHC-I expression is enhanced during pro-inflammatory stimuli, and this is associated with an increase in immunoproteasome-processed Ag peptides [104,210,213,215]. On the other hand, glial cells (both astrocytes and microglia) express MHC-I as well as MHC-II molecules, which recognize exogenous Ag peptides being processed by the immunoproteasome beyond, or in concert with, the classic lysosomal pathway, for subsequent CD4+ T-cells stimulation [216]. In detail, the induction of the immunoproteasome within DA neurons as well as microglia was recently related to α-syn immunogenic degradation and subsequent generation of self-Ag peptides for T-cell presentation by MHC-I [208,209,210]. Neuronal upregulation of Ag-loaded MHC-I can be induced by either microglial activation and subsequent IFN-γ release, or directly by the administration of α-syn or oxidant compounds in the absence of microglia or exogenously administered cytokines [210]. The cognate Ag/MHC-I complex exposed on the neuronal plasma membrane induces proliferation of CD8+ T-cells while triggering neuronal death via Fas/Fas ligand and perforin/granzyme pathways [210]. Moreover, despite not being endogenous to MHC-II-expressing cells, α-syn may be spread to and subsequently processed within glial cells for MHC-II display [209]. Thus, in glial cells, the immunoproteasome may also unconventionally process phagocytosed proteins to produce Ags which bind on MHC-II molecules, thus priming CD4+ Th lymphocytes and production of pro-inflammatory cytokines in the brain. This is also documented in in vivo AD models, where the immunoproteasome exacerbates Aβ-induced microglial activation and pro-inflammatory cytokines secretion, leading to a worse cognitive impairment compared with immunoproteasome-deficient mice [211]. However, it still remains to be investigated whether Ag peptides derived from Aβ are recognized by both CD4+ and CD8+ T cells as reported for α-syn [209].

The abovementioned concepts also imply a bidirectional communication between the CNS and immune periphery [74,104], which for reasons of synthesis are not discussed in detail herewith. In any case, it is intriguing that just as it occurs within the CNS milieu, a prion-like propagation of immunoproteasome-processed Ag peptides from CNS-derived prionoids may occur through the glymphatic pathway to reach antigen-presenting cells and T-cells within lymphoid organs. Once activated, immune cells may in turn travel back to the CNS parenchyma after crossing all CNS barriers [74,104]. In this way, an immunoproteasome-dependent generation of Ag-peptides deriving from misfolded or post-translationally-modified proteins is key to producing both pro-inflammatory and cytotoxic effects within brain cells. In this context, AGEs and AGEs-modified proteins activate RAGEs and synergize with pro-inflammatory cytokines to enhance immunoproteasome upregulation within astrocytes, microglia and neurons, while affecting standard UPS and autophagy activity as well. In fact, immunoproteasome hyper-activation within either astrocytes, microglia or neurons occurs through intracellular pathways which orchestrate autophagy and apoptosis such as PKC, JAK-STAT, Nf-Kβ, TLR4 and mTORC1 [104,105,106,170,205,217,218,219].

It is intriguing that besides standard UPS and autophagy, mTOR activation is bound to the replacement of standard- with immune-proteasome subunits, providing a possible link between concomitant autophagy and UPS alterations, bridging altered proteostasis with neuroinflammation (Figure 2) [74]. Autophagy constitutively operates in neurons as well as astrocytes and microglial cells, where it is key to degrading phagocytosed Aβ or α-syn while restraining pro-inflammatory cytokine release, apoptosis, and neurotoxicity [220,221,222]. Inflammatory stimuli which are known to induce the immunoproteasome may impair autophagy activity within either glial cells or neurons. For instance, cytokines such as tumor necrosis factor alpha (TNF-α) impair autophagy flux in microglia, while fostering microglia polarization towards the pro-inflammatory phenotype M1 [223]. Autophagy inhibition exacerbates TNF-α-induced M1 polarization, and remarkably, it is sufficient to trigger microglia activation toward M1 status while producing neurotoxicity [223]. Conversely, autophagy induction promotes microglia polarization toward the M2 phenotype, thus blunting inflammation and neurotoxicity [223]. In this context, AGE-modified misfolded proteins may play a key role, as RAGE activation within microglia impairs lysosome acidification and autophagy flux, while promoting microglia-mediated neuro-inflammation [224]. However, conflicting results still exist on the role of RAGE signaling upon autophagy activity. For instance, autophagy activation was reported to exert a protective role against RAGE-mediated Aβ accumulation and neurotoxicity [225], though it has also been associated with RAGE signaling and neurotoxic RAGE-related Aβ treatment [226]. Further studies are needed to elucidate such an issue. Nonetheless, it is conceivable that a vicious circle of inflammatory and oxidative events may further exacerbate alterations in protein clearance, thus magnifying the effects of undigested prionoids, both intracellularly and extracellularly.

## 6. Conclusions

Both UPS and autophagy are key to preventing protein misfolding, aggregation and toxicity, while autophagy may play a leading role in dealing with large protein aggregates that are resistant to the UPS. This is supposed to restrain the cell-to-cell, prion-like propagation of proteins acting as pathological seeds within neighbor and distant recipient cells. In keeping with this, two streams of interpretation exist concerning the role of protein extracellular release. On the one hand, this may occur as a compensatory mechanism to cope with cell survival by getting rid of potentially toxic intracellular substrates. In this scenario, cell-to-cell communication represents a natural mechanism that brain cells have conserved to communicate with each other in a common environment. This mechanism may be exploited either when sharing essential cell constituents or for signaling potentially threatening stimuli to other cells. Nonetheless, indigested and post-translationally modified proteins, which are spread to neighbor cells upon fusion of the autophagy compartments with the plasma membrane, may trigger detrimental effects bridging oxidative and inflammatory reactions. In this context, cell-to-cell propagation of prionoids may be more harmful than their occurrence within intracellular aggregates. Here, UPS and autophagy configure as sentinels operating at the crossroad between proteostasis, intracellular secretory/trafficking pathways, inflammation and immunity, as exemplified by the paradigm of AGE-RAGEs.

A number of molecular mechanisms operate at the crossroad of UPS and autophagy. This becomes evident by the effects sorted by the manipulation of one system upon the other’s activity, which witnesses mutual relationships occurring between the two cell-clearing pathways. Although we are just scratching the surface, it is now clear that autophagy and UPS operate in close collaboration, and early alterations in one system may progressively impinge on the other, to foster protein aggregation and toxicity in various neurological disorders. Thus, when investigating potential strategies aimed at preventing or counteracting proteotoxicity, the status of one system cannot be neglected when modulators of the other are employed. This is also key to dissecting any potentially different contributions of each pathway. This is best exemplified by mTOR inhibitors, which while being widely used as a strategy to rescue autophagy, may stimulate UPS-dependent protein degradation as well. To date, these concepts are still poorly addressed by experimental studies. Besides mTOR, a variety of molecular pathways such as TFEB, HSPA8-BAG3/BAG1 and GSk3β, do operate at the crossroad between autophagy and the UPS. All these pathways are presently explored as potential strategies in neurodegenerative proteinopathies Thus, we believe that further investigating mechanisms involved in the cross-talk between UPS and autophagy may improve current therapeutics of proteinopathies.

## Abbreviations

AD	Alzheimer’s Disease
PD	Parkinson’s Disease
HD	Huntington Disease
ALS	Amyotrophic Lateral Sclerosis
FTD	Frontotemporal Dementia
MSA	Multiple System Atrophy
α-syn	Alpha-Synuclein
Aβ	Amyloid-Beta
AβPP	Amyloid-Beta Precursor Protein
TDP-43	Tar-DNA-Binding Protein of 43 KDa
SOD	Cu/Zn Superoxide Dismutase
FUS	Fused In Sarcoma
UPS	Ubiquitin Proteasome System
UBQLNs	Ubiquilin Proteins
HDAC6	Histone Deacetylase 6
UBA	Ubiquitin-Associated
UBL	Ubiquitin-Ligated
UBA6	Ubiquitin-Activating Enzyme (E1)
BIRC6	Hybrid Ubiquitin-Conjugating Enzyme/Ubiquitin-Ligase (E2/E3)
GABARAP	GABA Type A Receptor-Associated Protein
METH	Methamphetamine
mTOR	Mammalian/Mechanistic Target Of Rapamycin
TFEB	Transcription Factor EB
FBXO7	F-box only protein 7
LAMP1	Lysosomal-Associated Membrane Protein 1
LC3	Microtubule-Associated Protein 1a/1b-Light Chain 3
HERC1	HECT and RLD Domain Containing E3 Ubiquitin Protein Ligase Family Member 1
HSPA8	Heat Shock Protein Family A (Hsp70) Member 8
HSPA1A	Heat Shock Protein Family A (Hsp70) Member 1A
BAG1/3	Bcl2-Associated Athanogene 1/3
DLB	Dementia with Lewy Bodies
PSP	Progressive Supranuclear Palsy
MPTP	1-Methyl-4-Phenyl-1,2,3,6-Tetrahydropyridine
6-OHDA	6-Hydroxydopamine
3-MA	3-Methyladenine
USP9X	Ubiquitin Specific Peptidase 9 X-Linked
APP	Amyloid Precursor Protein
GSK3β	Glycogen Synthase Kinase3 Beta
BACE2	Beta-Secretase 2
POLDIP2	DNA Polymerase Delta Interacting Protein 2
NUB1	Negative Regulator of Ubiquitin-Like Protein 1
USP14	Ubiquitin Specific Peptidase 14
HSC70	Heat Shock Cognate 71 Kda Protein (Hsc70), also known as HSPA8
EVs	Extracellular Vesicles
CSF	Cerebrospinal Fluid
CNS	Central Nervous System
AGEs	Advanced Glycation End Products
RAGEs	Receptors for Advanced Glycation End Products
PKC	Protein Kinase C
NF-kB	Nuclear Factor Kappa-Light-Chain-Enhancer of Activated B Cells
JAK2/STAT1	Janus Kinase 2/Signal Transducer and Activator of Transcription 1
Ag	Antigen
MHC-I	Major Histocompatibility Complex Type I
MHC-II	Major Histocompatibility Complex Type II
CD8+ T-cells	CD8 positive Cytotoxic T Lymphocytes
CD4+ Th cells	CD4 positive T Helper Lymphocytes
TLR4	Toll-Like Receptor 4
ULK1	Unc-51-Like Autophagy-Activating Kinase 1
